# The Glorious Sun-Shine

**Published:** 1859-11

**Authors:** 


### THE GLORIOUS SUN-SHINE.

Dr. Kane and his men, in their arctic voyage, had abundance of exercise, and the purest and most luscious air, nor were they exposed to malarias or miasm; in such a pure cold air there was no cause of noxious exhalations, and yet they had to contend against disease, in consequence of the mischievous effects of the absence of sun-shine for many weeks together.

The general reader may be familiar with the observation of an eminent merchant that his book-keepers soon became ill, and some of them died; the office being in a room where no sun-shine ever came; on changing to an upper room, the windows of which faced the sun, there was an immediate and permanent removal of the difficulty.

Dogs kept in dark, damp cellars become tuberculous in a few weeks, and die of consumption.

A New York merchant of wealth purchased a farm out west for a promising son; within a year he became unwell. Inquiries were made as to his sleeping-room, the answer was, that he had for his chamber a large upper room, well lighted. His sister paid him a visit, and soon observed that his clothing in his wardrobe was damp, while that in the drawers was actually moulded, when the fact presented itself that the room was on the north side of the house, overlooking an immense flat prairie, and that no ray of sun-shine ever entered from one year's end to another. He returned to New York and died of tubercular disease, which, with great certainty, was hastened, if not originated, by the unfortunate position of his chamber. The lesson is, that the family room, the sleeping apartment, the study, in short, any apartment which is occupied for the greater part of each twenty-four hours, should have its windows facing the south, as nearly as possible, so that the glad sun-shine may lighten it up, and keep it warm, and dry and pure.

